# Some Critical Reflections on the SEM-EDS Microanalysis of the Hydrotalcite-like Phase in Slag Cement Paste

**DOI:** 10.3390/ma16083143

**Published:** 2023-04-16

**Authors:** Yu Zhang, Karthikeyan Saravanakumar, Oğuzhan Çopuroğlu

**Affiliations:** Microlab, Section of Materials and Environment, Faculty of Civil Engineering and Geosciences, Delft University of Technology, 2628 CN Delft, The Netherlands; k.saravanakumar@student.tudelft.nl (K.S.); o.copuroglu@tudelft.nl (O.Ç.)

**Keywords:** hydrotalcite-like phase, accelerating voltage, standard-based and standardless microanalysis, compositional zonation

## Abstract

For a better understanding of the hydrotalcite-like phase with SEM-EDS microanalysis, the present research paid special attention to the data acquisition and interpretation of this technique. A lower Mg/Al ratio was obtained when using a higher accelerating voltage, and a beam energy of 10 kV was more appropriate than 15 kV for investigation when the slag rim was thin, to compromise to meet the requirements of obtaining an adequate overvoltage ratio and minimizing the interference. Additionally, it was noted that the Mg/Al ratio decreased from zones rich in hydrotalcite-like phase to zones rich in the C−S−H gel phase, and indiscriminately fitting scatter points selected from the slag rim would bias the Mg/Al ratio of the hydrotalcite-like phase. According to the standard-based microanalysis, it was concluded that the analysis total of the hydrates within the slag rim was in the range of 30–40%, lower than that located in the cement matrix. Besides the water chemically bound in the C−S−H gel phase, the hydrotalcite-like phase also contained a certain amount of hydroxide ions and chemically bound water.

## 1. Introduction

As a mature addition, ground-granulated blast furnace slag (slag for short) has been used as a supplementary cementitious material (SCM) in the cement industry for close to a century in Europe, North America, etc. [1,2,3,4]. The activation of slag particles depends mainly upon the breakdown of the slag network structure by OH^−^ ions, released from the hydration of cement clinkers. Secondary precipitations, e.g., the C−S−H gel phase and the hydrotalcite-like phase, originating from the hydration of the slag, have been identified [5]. These two phases are intermixed with each other, forming the so-called ‘inner’ products of slag within the original slag particle area (also called as the slag rim). The results from [6] pointed out that the blended mixture of the C−S−H gel phase and the hydrotalcite-like phase was formed by an in situ reaction between the surface of the unhydrated slag particles and the OH^−^ ions in the nearby pore solution. Based on FEI Nova NanoSEM 630 and FEI Talos F200X, the author in [7] put forward a hypothesis that the positively charged hydrotalcite-like phase layers were strongly attracted to the negatively charged C−S−H gel phase layers in the rim of the slag. On the other hand, instead of intimately mixing with each other, the results in [8] illustrated three distinct regions around unhydrated slag particles owing to the spatial zonation of the hydrotalcite-like phase, the C−S−H gel phase, and the Ca−Al phase. In a previous work [9], we also characterized the elemental compositions of the slag rim in a 40-year-old slag concrete sample. A clear zonation phenomenon was observed, and it was concluded that the formation and distribution of the hydrates within the slag rim were determined by the original slag particle size and the low mobility of magnesium.

Hydrotalcite, also referred to as Layered Double Hydroxides (LDHs), is well recognized as an anionic clay because of its strong anion-exchange property [10]. It can be expressed as the general formula $\left[M_{1-x}^{2+}M_{x}^{3+}{\left(OH\right)}_{2}\right]A_{x/n}^{n-}∙yH_{2}O$, where *M*^2+^ and *M*^3+^ are divalent and trivalent cations, respectively, *A^n−^* is the anion fixed in the interlayer with a valence of n, and x equals *M*^3+^/(*M*^2+^ + *M*^3+^) in molar fraction, ranging from 0.17 to 0.33 [11,12,13]. In naturally occurring hydrotalcite, the most common anion detected is carbonate (CO_3_^2−^). Nonetheless, hydrotalcite can accommodate various anionic species in the interlayer without restriction. Meanwhile, water molecules occupy the free space of the interlayer via hydrogen bonding [12,14,15].

In cement chemistry, hydrotalcite represents a group of phases, e.g., Mg-Al LDHs formed in the slag-containing paste and Ca-Al LDHs, the hydration products of tricalcium aluminate (C_3_A) [16]. The present research mainly focuses on Mg-Al LDHs, the main precipitation sourced from the hydration of the slag. Two parameters should be addressed when referring to hydrotalcite. One is the Mg/Al (atomic) ratio and the other one is the interlayer anion, the amount of which is dependent on the Mg/Al ratio for electro-neutrality. In general, the Mg/Al ratio of hydrotalcite can be obtained from the slope of the regression line when fitting the scatter plot of Mg/Si against Al/Si, by means of scanning electron microscopy (SEM), e.g., in [17,18,19,20,21], or transmission electron microscopy (TEM), e.g., in [22,23,24]. According to the results in [25], the Mg/Al ratio of hydrotalcite formed in the slag cement and alkali-activated slag pastes was not fixed and differed over a wide range. In most cases, the value was less than 2.0, the probable minimum value for natural and synthesized hydrotalcite [26]. Thus, the term ‘hydrotalcite-like phase’ is used to name it in cementitious systems. As for its interlayer anion, it was confirmed to be hydroxide (OH^−^) under a sealed curing condition, through evolved gas analysis [22].

As mentioned, through backscattered electron (BSE) in tandem with energy-dispersive spectrometry (EDS), SEM has made a considerable contribution to understanding the hydrotalcite-like phase formed in slag cement and alkali-activated slag systems. On the other hand, SEM is probably the most widely misused technique in cement science, and a very high proportion of published articles provides no useful or misleading information. For example, an accelerating voltage of 15 kV was frequently reported to perform point analysis/line scanning at the slag rim [18,19,20,21]. However, little research noted the mismatch between the interaction volume under this accelerating voltage and the thickness of the slag rim. Its influence on the determined Mg/Al ratio was therefore scarce. Additionally, owing to the short curing age of most experiments carried out in the laboratory, the effect of the compositional zonation within the slag rim on determining the Mg/Al ratio was also unknown. Therefore, for the better understanding of the hydrotalcite-like phase with SEM-EDS microanalysis, the authors in the present paper laid emphasis on, e.g., the influence of the accelerating voltage on determining the Mg/Al ratio, its analysis total based on the quantitative microanalysis, etc. It is believed that the results obtained in the current study can provide guidance for the best use of SEM including data acquisition and interpretation and contribute to the understanding of the hydrotalcite-like phase formed in various cementitious systems.

## 2. Materials and Methodology

### 2.1. Sample Information

Two slag concrete samples (A and B) collected from a field in the Netherlands were studied in the paper, the service lives of which were approximately 40 years. A brief introduction of them is given in Table 1.

To characterize the hydrates formed in these two samples, thermogravimetric analysis (TGA) was performed. Slices taken from each specimen were placed in isopropanol solution to extract the free water. To obtain particles with a diameter < 63 μm, the fragments were crushed, ground with a mortar, dried in an oven of 40 °C, and sieved manually. TGA was carried out on a Netzsch STA 449 F3 Jupiter coupled with a mass spectrometer (MS) Netzsch QMS 430 C. The emission of H_2_O and CO_2_ after heating was thus identified. Sample powders of about 50 mg were heated at a rate of 10 °C per minute from 40 to 800 °C in an argon environment.

**Figure 1:**
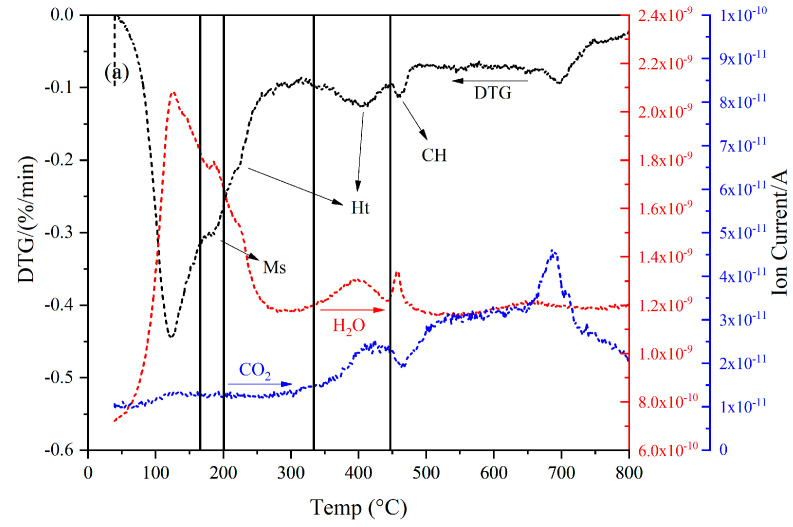

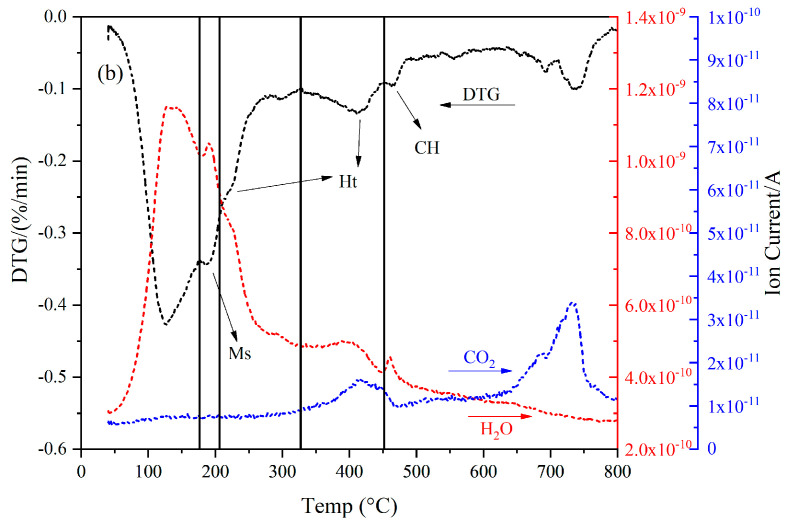
The DTG results, H_2_O and CO_2_ MS curves of (**a**) sample A and (**b**) sample B, respectively. CH: portlandite; Ht: hydrotalcite-like phase; Ms: calcium monosulfoaluminate.

The DTG (derivative thermogravimetric) results of these two samples are shown in Figure 1. The main hydrates formed in these two samples were similar. The peak between 400 and 500 °C, originating from the dehydroxylation of portlandite, almost disappeared. The peaks located at ~250 and 350 °C indicated the precipitation of the hydrotalcite-like phase. The peak at 100–150 °C suggested the presence of the C−S(A)−H gel phase. The shoulder at ~200 °C implied the formation of calcium monosulfoaluminate, from the transformation of ettringite over time [4].

As for the H_2_O and CO_2_ MS curves, the mass loss before 300 °C was mainly due to the evaporation of H_2_O. On the other hand, the decomposition of the carbonate-type phases dominated the mass loss after 500 °C, as verified by the CO_2_ MS curve. These phases can be ascribed to the carbonation of specimens after such a long service life. In particular, the peak at ~700 °C can be considered as the decomposition of calcite. Meanwhile, both H_2_O and CO_2_ were released from 350 to 450 °C. In other words, some hydroxide ions initially fixed in the interlayer space of hydrotalcite-like phase were exchanged for carbonate ions after ~40 years of natural exposure.

### 2.2. SEM-EDS Microanalysis

All samples of approximately 6 mm in height were cut and immersed in isopropanol solution to stop hydration. Afterwards, the samples were dried in a 40 °C oven and then impregnated with epoxy resin. Polished sections down to 0.25 μm were prepared. Finally, the well-polished samples were carbon coated in a Leica EM CED 030 carbon evaporator.

An FEI Quanta FEG 650 ESEM equipped with a silicon drift Thermo Fischer EDS detector was employed in a high vacuum chamber condition. All microanalysis was performed at a working distance of 10 mm. For other instrumentally related parameters, the readers can refer to [27]. Hydrates in the rim of the slag were characterized with both standard-based (quantitative) and standardless (semi-quantitative) microanalysis. Because the current research concentrated on the precipitations within the slag rim (the hydrotalcite-like phase in the particle), only four elements were involved, i.e., Ca, Si, Al, and Mg. Thus, four compounds (see Table 2) were selected as quantitative microanalysis standards from a commercial mineral standard mount (MINM25-53 Serial BW from Astimex Scientific Ltd., Toronto, ON, Canada). As for the other trace elements, e.g., Ti, Mn, Fe, K, Na, S, etc., they were not considered here due to their significantly low mass percentage [27]. For standard-based (std.-based) microanalysis, the calibration to the reference standard mounts was modeled with NIST DTSA-II Microscopium software [28,29]. The unknown spectra were quantified by the k-ratio fitting routine, using the ratio of the intensity of the X-ray peaks in unknown and standards of known compositions and by applying matrix correction procedures. Additionally, oxygen was quantified stoichiometrically. As for standardless microanalysis, it relied on the internal standards provided by the commercial software Pathfinder (Thermo Fisher Scientific). For the detailed introduction to the quantitative microanalysis process, the readers can also refer to [27]. It is worthwhile to mention here that the results of the std.-based microanalysis are mainly presented in Section 3.2.

## 3. Results and Discussion

### 3.1. The Thickness of the Slag Rim and the Interaction Volume of the Electron

#### 3.1.1. The Thickness of the Slag Rim

**Figure 2:**
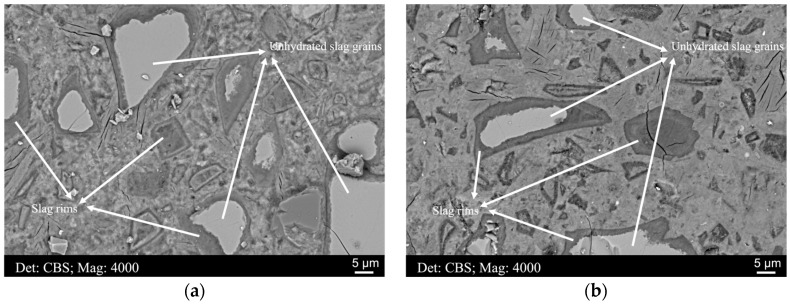
The microstructures of (**a**) sample A and (**b**) sample B, respectively.

During the hydration of slag grains, secondary formations, including the hydrotalcite-like phase and the C−S−H gel phase, are precipitated within the original slag particle areas, forming dark rims around unhydrated slag grains [5]. The slag rim thickens accompanied by the continuous dissolution of the unhydrated slag particles and the precipitation of hydrates with the extension of the curing age. Generally, the thickness of the slag rim depends on several factors, e.g., the reactivity of the slag, the slag replacement level, the pH of the pore solution, the curing age, etc. Figure 2a,b illustrate the representative microstructures of samples A and B, respectively. After such a long hydration time (decades), fully and partially hydrated slag grains were observed frequently across the matrix, and the thickness of the slag rim fluctuated considerably, in the range of 1~10 μm.

#### 3.1.2. Accelerating Voltage and Interaction Volume

For the work of electron microscopy, the electrons accelerated by the instrument experience a series of collisions with atoms on the surface of the material. The volume in which these collisions occur is considered as the ‘interaction volume’. The interaction volume of cementitious materials, the elements of which present a comparatively low atomic number, is about a few microns [30].

**Figure 3:**
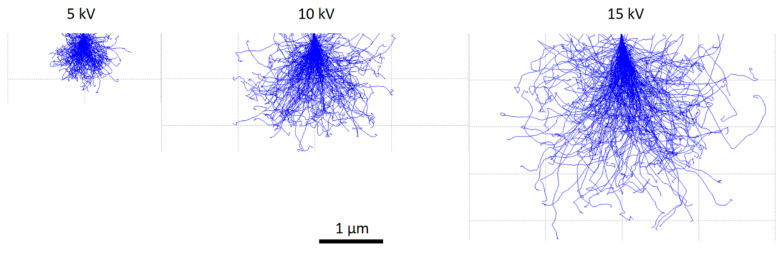
Monte Carlo simulation of the penetration of 200 electrons accelerated at 5, 10, and 15 kV into the slag rim.

The interaction volume targeted at the slag rim was estimated by Monte Carlo simulation in CASINO version 2.48 software (https://www.gegi.usherbrooke.ca/casino/, accessed on 4 July 2007) at accelerating voltages of 5, 10, and 15 kV, respectively (For some useful tutorials, please refer to https://www.gegi.usherbrooke.ca/casino/tutorial/tutorial_frames.html, accessed on 4 July 2007). Figure 3 illustrates the maximum penetration depth of the electron trajectories into a hypothetical slag rim. The lateral dimension was close to the depth of the interaction volume [31]. As can be seen, an increase in the penetration depth from ~0.5 μm at 5 kV to ~3.0 μm at 15 kV and a corresponding rise in the interaction volume from less than 0.5 μm^3^ at 5 kV to ~25.0 μm^3^ at 15 kV was determined by the simulation.

Therefore, one should be careful to select the accelerating voltage to perform EDS analysis when comparing the thickness of the slag rim with the lateral dimension of interaction volume. A 15 kV or higher accelerating voltage is irrational for samples with a thin slag rim (<~3.0 μm), as a large quantity of interference from both the unhydrated slag grain and the surrounding cement matrix would be incorporated into the analysis. As for 10 kV, the researchers should also be very careful as the lateral dimension of the interaction volume at this voltage is slightly greater than 1 μm, also greater than the thickness of some rims formed around very large slag particles (Figure 2).

On the other hand, the overvoltage ratio is another factor that should be noted when determining the accelerating voltage. It is the ratio of the beam energy E_0_ to the ionization energy E_i_, in the form of E_0_/E_i_. In the study with an electron microscope, E_0_ is the accelerating voltage, and E_i_ is the considered X-ray energy in keV. For optimized SEM-EDS microanalysis, the typical overvoltage ratio should be at least 2 for the highest energy line and no more than 20 for the lowest energy line of interest, to properly measure the element with the highest X-ray energy (K*α*) [31]. As calcium Ca (K*α* = 3.692 keV) is usually the element with the highest X-ray energy in the rim, a beam energy of at least 7.5 kV is required.

#### 3.1.3. The Influence of the Accelerating Voltage on Determining the Mg/Al Ratio of the Hydrotalcite-like Phase

As discussed in Section 3.1.2, there is a tradeoff for choosing the accelerating voltage between the requirements of obtaining an adequate overvoltage ratio and minimizing the interference. In this section, 10 and 15 kV were adopted to give a direct demonstration of the influence of the accelerating voltage on determining the Mg/Al ratio of the hydrotalcite-like phase.

**Figure 4:**
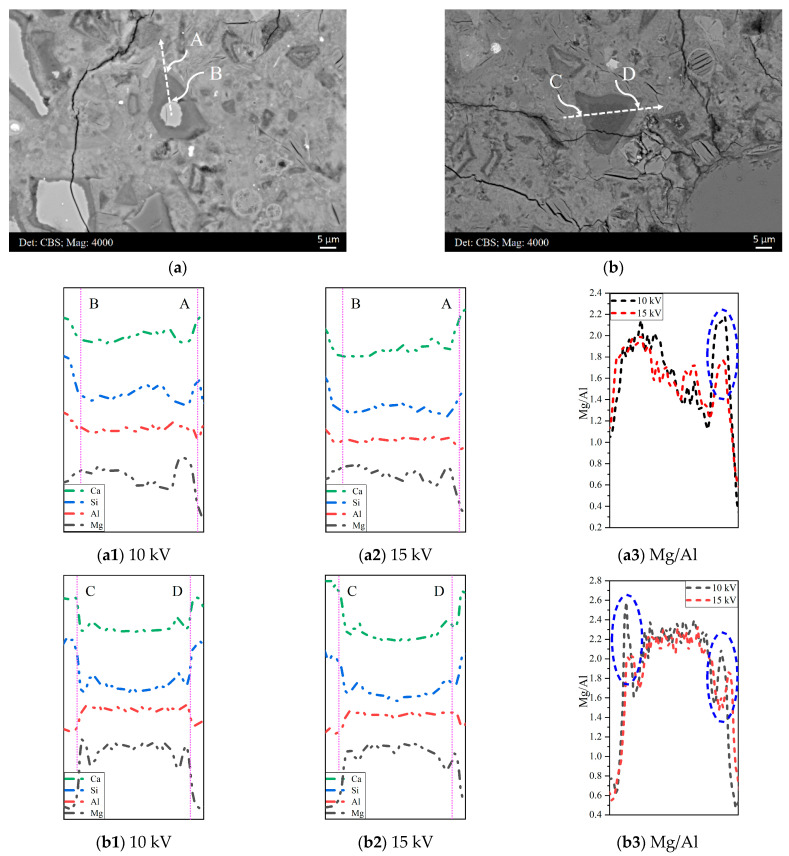
(**a**,**b**): Two representative BSE micrographs of the microstructure of sample B. EDS line scan-profiles of the elements (Ca, Si, Al, and Mg) along the dissection lines (**a1**,**a2**): A-B; (**b1**,**b2**): C-D under 10 and 15 kV accelerating voltages, respectively. The Mg/Al atomic ratio down the lines (**a3**): A-B and (**b3**): C-D.

Figure 4a,b display two representative BSE micrographs of the microstructure of sample B. EDS line scan-profiles of the elements (Ca, Si, Al, and Mg) along the dissection line A-B under 10 and 15 kV accelerating voltages and the corresponding Mg/Al atomic ratio are shown in Figure 4(a1,a2,a3), respectively. Similarly, the results along the dissection line C–D are displayed in Figure 4(b1,b2,b3), correspondingly. In general, the Mg/Al atomic ratio varied slightly in regions where the concentrations of Mg and Al were stable, e.g., the unhydrated slag particle area along line A-B and the middle region along line C-D. Although a larger interaction volume was generated and more interference was incorporated by the higher accelerating voltage, it did not exert a significant impact on the Mg/Al ratio of these areas.

However, a clear fluctuation regarding the Mg/Al ratio was observed near the border connected to the cement matrix under different accelerating voltages. A higher accelerating voltage led to a reduced Mg/Al ratio, as circled in Figure 4(a3,b3). As shown in the EDS line scan-profiles (Figure 4(a1,a2,b1,b2)), the Mg concentration presented as a peak in the border of the slag rim, whereas it decreased in both sides, especially in the side of the cement matrix, where the Mg content decreased to null approximately. On the other hand, the reduction in the Al concentration in both sides was not as significant as that of Mg, and it even levelled off in the side of the ‘inner’ products. Thus, for a larger interaction volume generated by a higher accelerating voltage, the interference from the cement matrix (nearly null Mg content) would be incorporated, and the average reduction in the Mg concentration would be more remarkable, resulting in a lower Mg/Al ratio in the border of the slag rim.

Note that for the slag-containing systems hydrated for a short period, commonly less than one year for most experiments conducted in the laboratory, the thickness of the slag rim is even smaller, and the EDS scatter points are inevitably selected close to the border. To minimize the interference from the surroundings, a beam energy of 10 kV is more appropriate than 15 kV.

### 3.2. Std.-Based and Standardless Microanalysis

In this section, a beam energy of 10 kV was adopted as the accelerating voltage for both std.-based and standardless microanalysis. About 50 scatter points located at the slag rim were analyzed for each mixture, and the average values are reported in Table 3.

Due to the presence of the chemically bound water in the C−S−H gel phase and the hydrotalcite-like phase, the analysis total of each point could not reach 95–105%, as hydrogen was not taken into the calibration. Compared to the analysis total of the hydrates in the cement matrix, which ranged from 65 to 85% [32,33], the analysis total of the hydrates within the slag rim was much lower, in the range of 30–40%. It was reasonable, as aside from the water chemically bound in the C−S−H gel phase, the hydrotalcite-like phase also contained a certain amount of hydroxide ions and chemically bound water. Furthermore, note that these two samples experienced some extent of carbonation (Figure 1), and carbon (C) was not considered in the study. Additionally, it was found that the Mg/Al atomic ratio obtained from these two methods was somehow different. In the works of [34,35,36], the authors elaborated the differences between std.-based and standardless EDS microanalysis, which were most likely caused by the uncertainties in the k-ratio protocol of the standardless microanalysis.

### 3.3. Compositional Zonation within Slag Rim

As mentioned earlier, the hydrotalcite-like phase is blended with the C−S−H gel phase, forming the so-called ‘inner’ products of the slag. Initially, the ‘inner’ products mainly consist of a virtually featureless shell of gel. This gel may be homogeneous and intermediate in composition between the C−S−H gel phase and the hydrotalcite-like phase [37]. With the hydration proceeding, a distinct increase in the Mg concentration and a decrease in the Ca content were found in the rim of the slag (see Figure 4). Meanwhile, clear compositional zonation was identified within the slag rim over time [8,9]. Under this circumstance, the C−S−H gel phase and the hydrotalcite-like phase were separated from each other as much as possible, and scatter points could be targeted only at the hydrotalcite-like phase.

**Figure 5:**
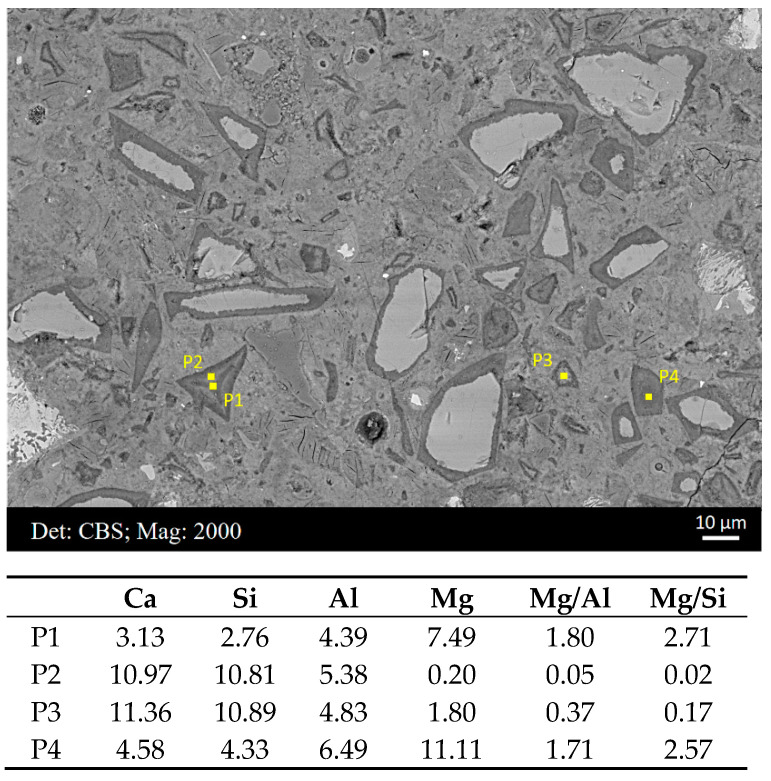
Some typical scatter points extracted from the point analysis of sample B, with elemental compositions exhibited in the table below.

In Figure 2a,b, clear compositional zonation was observed in the rim of sample A and B, respectively. Thus, EDS scatter points were targeted at the regions rich in the hydrotalcite-like phase (relatively dark coloration with a low mean atomic number) and the C−S−H gel phase (relatively light coloration with a comparatively higher mean atomic number), separately. Some typical scatter points are illustrated in Figure 5, with elemental compositions exhibited in the table below. Points 1 and 4 were located at regions rich in the hydrotalcite-like phase, and the Mg/Al ratio was in the range of 1.5–2.0. Points 2 and 3 were in areas accumulated with the C−S−H gel phase, and it was noted that the Mg content was reduced to approximately null in these regions.

**Figure 6:**
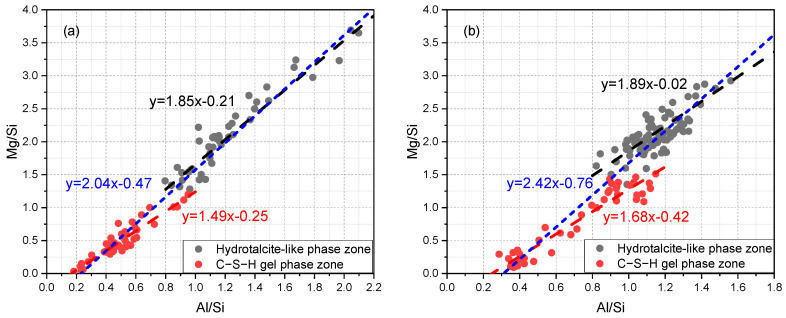
The scatter plots of Mg/Si vs. Al/Si in the molar ratio of (**a**) sample A and (**b**) sample B, respectively.

Moreover, the scatter plots of the Mg/Si vs. Al/Si of sample A and B are given in Figure 6a,b, respectively. It is recognized that the slope of regression line indicates the Mg/Al atomic ratio of the hydrotalcite-like phase [30,38,39]. The points along the established trend line (or ‘tie-line’) suggest the binary mixture of the hydrotalcite-like phase and the C−S−H gel phase. As can be seen, the scatter points selected from the regions abundant in the hydrotalcite-like phase were in the upper part of graph, characterized by Mg/Si > 1.0 or even 1.5. On the contrary, the other points representative of areas rich in the C−S−H gel phase were situated in the lower part, and the prominent feature was Mg/Si < 1.0. Note that the fitted Mg/Al ratio decreased from a zone rich in the hydrotalcite-like phase to a zone rich in the C−S−H gel phase, as the Mg concentration decreased significantly. At the same time, regression lines fitting all points were also present in the graphs (blue dashed line). Apparently, an indiscriminate fitting would bias the Mg/Al ratio of the hydrotalcite-like phase. Researchers should be aware of the spatial distribution of the hydrates when the zonation phenomenon occurs in the rim and choose the right regions to conduct point analysis.

## 4. Conclusions

The authors in the present paper provided some critical reflections on characterizing the Mg/Al atomic ratio of the hydrotalcite-like phase formed in the cement–slag system through SEM-EDS microanalysis. The main conclusions were drawn as follows:

1. During microanalysis, the beam energy of 10 kV was more appropriate than 15 kV for samples with a thin slag rim to compromise to meet the requirements of obtaining an adequate overvoltage ratio and minimize the interference.
2. A lower Mg/Al ratio was obtained when a higher accelerating voltage was employed. The effect was especially distinct in the border of the slag rim where there was a significant fluctuation in the Mg content.
3. The analysis total of the hydrates within the slag rim was in the range of 30–40%, lower than that in the cement matrix. Aside from the water chemically bound in the C−S−H gel phase, the hydrotalcite-like phase also contained a certain amount of hydroxide ions and chemically bound water.
4. It was noted that the Mg/Al ratio decreased from zones rich in the hydrotalcite-like phase to zones rich in the C−S−H gel phase. Indiscriminately fitting scatter points selected from the slag rim would bias the Mg/Al ratio of the hydrotalcite-like phase.

These conclusions based on the cement–slag system can also be extended to characterize the hydrotalcite-like phase formed in other cementitious systems, e.g., alkali-activated slag paste, dolomite-containing paste, etc. Meanwhile, as hydrotalcite-like can bind various anionic species in the interlayer space under various aggressive environments, it is also believed that the results obtained in the current study can provide guidelines for the best use of SEM to investigate the correlation between the hydrotalcite-like phase and the durability-related issue of the systems.

## Figures and Tables

**Table 1:** Descriptive information of the slag concrete samples studied in the paper.

Sample	
A	The sample was sourced from a parking garage built around 1980. It was in Jupiterstraat, Hoofddorp. The cement was partially replaced by blast furnace slag.
B	The sample was collected from a wind deflection screen near Calandbrug, Europoort Rotterdam (Port of Rotterdam), which was built in 1985. The cement type was reported as CEM III/B.

**Table 2:** Compounds used as the standards for the quantitative EDS microanalysis.

Target Element	Mineral	Composition
Ca	Calcite	CaCO_3_
Si	Quartz	SiO_2_
Al	Albite	NaAlSi_3_O_8_
Mg	Dolomite	MgCa(CO_3_)_2_

**Table 3:** Results based on the std.-based and standardless EDS microanalysis.

	Mg/Al		% Analysis Total	
	Std.-Based	Standardless	Std.-Based	Standardless
Sample A	1.74	1.86	33.4	100.0
Sample B	1.79	1.91	31.2	100.0

## Data Availability

The data presented in this study are available on request from the corresponding author. The data are not publicly available due to privacy.
